# *Streptococcus uberis* and *Staphylococcus aureus* forefoot and blood stream co-infection in a haemodialysis patient: a case report

**DOI:** 10.1186/s12882-015-0069-6

**Published:** 2015-05-28

**Authors:** Christine Valentiny, Harald Dirschmid, Karl Lhotta

**Affiliations:** Department of Nephrology and Dialysis, Academic Teaching Hospital Feldkirch, Carinagasse 47, A-6800 Feldkirch, Austria; Institute for Pathology, Academic Teaching Hospital Feldkirch, Feldkirch, Austria

**Keywords:** Haemodialysis, Infection, Streptococcus

## Abstract

**Background:**

*Streptococcus uberis,* the most frequent cause of mastitis in lactating cows, is considered non-pathogenic for humans. Only a few case reports have described human infections with this microorganism, which is notoriously difficult to identify.

**Case presentation:**

We report the case of a 75-year-old male haemodialysis patient, who developed a severe foot infection with osteomyelitis and bacteraemia. Both *Streptococcus uberis* and *Staphylococcus aureus* were identified in wound secretion and blood samples using mass spectrometry. The presence of *Streptococcus uberis* was confirmed by superoxide dismutase A sequencing. The patient recovered after amputation of the forefoot and antibiotic treatment with ampicillin/sulbactam. He had probably acquired the infection while walking barefoot on cattle pasture land.

**Conclusion:**

This is the first case report of a human infection with *Streptococcus uberis* with identification of the microorganism using modern molecular technology. We propose that *Staphylococcus aureus* co-infection was a prerequisite for deep wound and bloodstream infection with *Streptococcus uberis*.

**Electronic supplementary material:**

The online version of this article (doi:10.1186/s12882-015-0069-6) contains supplementary material, which is available to authorized users.

## Background

End-stage renal disease is a state of severe immunodeficiency and dialysis patients are prone to infectious complications. We describe a haemodialysis patient who acquired a wound infection and bacteraemia with *Streptococcus uberis*, which is a microorganism that causes mastitis in cows but is considered non-pathogenic in humans. We propose that invasive infection was facilitated by a co-infection with *Staphylococcus aureus*. In addition, we provide an overview of case reports of *S. uberis* infections in humans.

## Case presentation

A 75-year-old male Caucasian haemodialysis patient presented with painful swelling and redness of his right forefoot. He had a fever of 39.0 °C and hypotension. C-reactive protein was elevated at 300 mg/l and interleukin-6 was 1120 pg/ml. His leukocyte count was 20 G/l. One week earlier he had fallen and suffered subluxation of the second and third metatarsophalangeal joints of the right foot. Two days later a haemorrhagic plantar bulla developed. At presentation a secreting ulcer was visible at the region of the second and third metatarsophalangeal joints. In a swab specimen taken from wound secretion methicillin-susceptible *S. aureus, Acinetobacter baumannii, Proteus vulgaris, S. uberis and Clostridium perfringens* were cultured. A concomitant blood culture sample was positive for *S. aureus and S. uberis.*

The first toe had been amputated 4 months earlier because of staphylococcal osteomyelitis. The previous year he had had an abscess on the left forefoot with septicaemia caused by methicillin-resistant staphylococci, and this had required surgical drainage.

Clinically significant peripheral arterial vascular disease was excluded. The next day an urgent transmetatarsal amputation of the right forefoot was performed. A deep wound swab led to growth of *S. aureus* and *S. uberis*. Histology showed a fistulating ulcer affecting the bones, putrid thrombophlebitis and gangrene of the plantar soft tissue. The patient was treated with intravenous ampicillin 2 g/sulbactam 1 g after each haemodialysis for the following 3 weeks. His clinical condition and inflammatory parameters rapidly improved. Wound healing was protracted and a secondary infection with *S. aureus* was treated with linezolid. Six weeks after the incident the patient had completely recovered.

The isolate of *S. uberis* was initially identified using matrix-assisted laser desorption ionisation time-of-flight mass spectrometry (MALDI TOF) (MALDI Biotyper©Microflex LT, Bruker Daltonics, Bremen, Germany). The *S. uberis* isolate obtained from the blood culture was sent to the German National Reference Centre for Streptococci, where the correct classification was confirmed by sequencing the superoxide dismutase A gene.

## Discussion

To the best of our knowledge this is the first description of *S. uberis* infection in a haemodialysis patient. *S. uberis* is the most common pathogen causing mastitis in cows worldwide [1]. *S. uberis* causes painful inflammation of the udder and decreases the quantity and quality of milk production. Because of antibiotic treatment of cows, *S. uberis* isolates with decreased susceptibility to penicillin, macrolides, aminoglycosides and clindamycin are increasingly reported [2]. *S. uberis* infection of cattle usually occurs after environmental exposure. One study showed *S. uberis* in 63 % of environmental samples such as water, soil, plant matter and hay [3]. In addition, 23 % of 104 fecal samples were positive for *S. uberis*, with the highest proportion observed during the summer grazing season. Therefore, fecal shedding is probably the main source of *S. uberis* infections in cattle.

*S. uberis* is considered non-pathogenic to humans. The species difference in pathogenicity is at least partly explained by the fact that *S. uberis* plasminogen activator (SUPA) activates bovine, but not human, plasminogen [4]. Activation of the plasminogen system causes degradation of the extracellular matrix and facilitates spreading of bacteria and invasive infection.

*S. uberis* belongs to the family of viridans streptococci. This pathogen has rarely been identified in human clinical microbiological samples. *S. uberis* has been reported as a cause of urinary tract infection. Gülen et al. described seven cases out of 148 culture-positive urinary tract infections from patients living in a rural Turkish region [5]. The authors speculate that patients became infected through frequent contact with cows and milk. One other case of S. uberis urinary tract infection was described by Lazinska et al. in 47 urine samples from 42 renal transplant recipients [6]. Unfortunately, clinical presentation, treatment and outcome in patients with *S. uberis* urinary tract infection have not been specified in detail. *S. uberis* has also been found to be a cause of bacterial pneumonia. Sarkar et al. described three cases of pneumonia with positive blood cultures [7]. All three patients recovered with iv penicillin therapy. Murdoch and MacFarlane described *S. uberis* as the cause of abscess formation after a scalp laceration from a horse kick in a child [8]. The child recovered after drainage and antibiotic therapy with amoxicillin and cotrimoxazole. Another case report described gonarthritis and soft tissue abscess caused by *S. uberis* [9]. The patient was treated with surgical drainage and cefuroxime, clindamycin and penicillin. In addition, endocarditis in a child with Down syndrome and an atrioventricular canal has also been reported [10]. A case of postoperative *S. uberis* endophthalmitis after phacoemulsification and vitrectomy for proliferative diabetic nephropathy has been described in a patient with diabetes and end-stage renal disease on peritoneal dialysis [11]. Sanches et al. reported a rancher, in whom *S. uberis* was identified from a hepatic abscess [12]. Table 1 shows previous reports of *S. uberis* infection. In summary, including our case we were able to identify 17 cases of *S. uberis* infection. In eight of these cases direct or indirect contact with cattle or milk and dairy products was suspected.

**Table 1:** Chronological list of case reports of *S. uberis* infection

Author, year	Patients	Type of infection	Diagnostic test^a^	Treatment	Outcome
Sarkar, 1989 [7]	3 healthy adults	pneumonia, bacteraemia	API 20 STREP	iv penicillin	recovered
Sanchez,1991 [12]	healthy adult (rancher)	hepatic abscess	VITEK	drainage, ampicillin	recovered
Murdoch, 1997 [8]	9-year-old child	abscess after scalp laceration	API 20 STREP	drainage, amoxicillin, cotrimoxazole	recovered
Bouskraoui, 1998 [10]	11-month-old child with Down syndrome, atriventricular canal	endocarditis	not specified	amoxicillin, clavulanic acid, gentamiycin	recovered
Lazinska, 2005 [6]	renal transplant recipient	urinary tract infection	API 20 STREP	not specified	not stated
Huang, 2007 [19]	2-year-old child, acute myeloid leukaemia	bacteraemia	not specified	not specified	not stated
Kessel, 2008 [9]	healthy adult	gonarthritis, muscular abscess	VITEK	surgery, cefuroxime, clindamycin, penicillin	impaired joint mobility
Velez-Montoya, 2010 [11]	adult, diabetes, peritoneal dialysis	postoperative endophthalmitis	not specified	intravitreal and iv ampicillin	blind in one eye
Gülen, 2013 [5]	healthy, age not specified	urinary tract infection (7 out of 148 samples)	conventional phenotyping BBL Crystal™	not specified	not stated

^a^Manufacturers of diagnostic tests: API 20 STREP and VITEK are provided by BioMerieux, France, and BBL Crystal™ is provided by Becton Dickinson and Company, USA

Our case is exceptional in several respects. First, to the best of our knowledge this is the only case in which the presence of S. uberis in a human clinical sample was confirmed by modern methodology, including MALDI TOF and superoxide dismutase A gene sequencing. These tests are currently the best methods for unequivocal classification of *S. uberis* [13, 14]. *S. uberis* is notoriously difficult to identify. In a study of 1227 *S. viridans* clinical human samples seven were initially classified as *S. uberis* [15]. However, in retrospect, using newer technology all of these samples were reclassified as *Globicatella sanguinis* [16]. In addition, *S. uberis* and enterococci appear to be difficult to distinguish by phenotypical testing only [17]. In the case reports listed in Table 1 diagnosis of *S. uberis* was established by phenotypical testing. Some of these infections might have been misclassified as previously suspected [18].

Second, this is the only report of *S. uberis* and *S aureus* co-infection requiring an amputation. Thirdly, to the best of our knowledge concomitant *S. uberis* wound infection and bacteraemia have also not been previously reported. In addition, our patient had deep wound infection with osteomyelitis and bacteraemia caused by *S. uberis* and *S. aureus* coinfection. He also had a mixed wound infection including several other bacteria that were found in superficial wound secretion. From a clinical point of view the exact pathogenetic roles of *S. uberis* and *S. aureus* are difficult to determine in our case. Because *S. aureus* is a highly pathogenic and tissue-invasive bacteria, we speculate that it was the primary and leading pathogen and facilitated subsequent invasion by *S. uberis*. Whether *S. uberis* alone would have been able to cause the severe infection is unknown but we believe this is unlikely. Notably, none of the other case reports mentioned co-infection with other more aggressive bacteria. Fortunately, *S. uberis* and *S. aureus* that were present in the deep wound and blood were susceptible to all tested antibiotics and the patient quickly recovered under ampicillin/sulbactam treatment. In case of a difference in antibiotic susceptibility between them we would have chosen an antibiotic combination therapy to target both organisms.

Notably, our case of a haemodialysis patient with wound infection and bacteraemia, a case of a peritoneal dialysis patient with endophthalmitis and a case of a renal transplant patient with urinary tract infection suggest that patients on renal replacement therapy are particularly susceptible to *S. uberis* infection [6, 11].

The last remaining question is how the patient acquired this rare infection. He had suffered two additional serious foot infections in the preceding year. The patient was a staunch barefoot walker and had calloused feet with multiple lacerated wounds on his soles. During the summer months he used to hike barefoot across mountain pastures. Summer is also the cattle grazing season, when alpine pastures are densely populated by cows. We believe that the patient inadvertently stepped in one of the innumerable cow pats that cover alpine pastures, thereby translocating the cow's faecal shedding into the cutaneous clefts. His fall with subluxation of the toe may have caused some of this material to translocate into deeper tissues, where it served as a nidus for the infection.

## Conclusion

In summary, we report the first case of *S. uberis* osteomyelitis and wound and bloodstream infection in a haemodialysis patient. This is the only case of a human infection that was diagnosed by modern microbiological methodology. Invasive infection was most likely facilitated by *S. aureus* co-infection. We recommend that immunocompromised patients should not to walk barefoot over cattle grazing pastures.

## Consent

Written informed consent was obtained from the patient for publication of this Case report. A copy of the written consent is available for review by the Editor of this journal.
